# Peripheral tolerance induced by apoptotic cells and PD-1+ CD8 T cells

**DOI:** 10.1186/ar3635

**Published:** 2012-02-09

**Authors:** Hirotaka Kazama, Tomonori Iyoda, Satoko Yokoyama, Kayo Inaba, Thomas A Ferguson, Shin Yonehara

**Affiliations:** 1 Department of Biostudies, Kyoto University Graduate School, Kyoto 606-8501 Japan; 2 Department of Ophthalmology and Visual Science, Washington University School of Medicine, MO 63110 USA

## 

**Figure 1:**
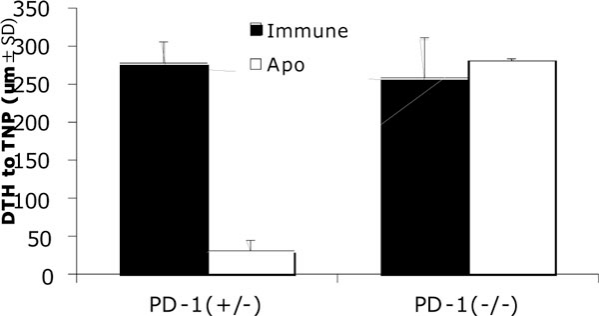
**PD-1 is essential for tolerance induced by apoptotic cells**. TNP-apoptotic cells were injected intravenously into PD-1 hetero- or homo- deficient mice. The mice were immunized with TNP (Filled bar) or preconditioned with apoptotic cells before immunization with TNP (open bar).

Self tolerization in peripheral is critical to prevent autoimmune diseases including arthritis and here we focus on the role of PD-1 in tolerance induction against the antigen associated with apoptotic cellsdelivered intravenously (i.v.). We accessed delayed type hypersensitivity (DTH) reaction against hapten (TNP) as antigen specific immune response, in which the injection of TNP-apoptotic cells i.v. suppressed DTH in wild type mice but we found not in PD-1 KO mice (Figure 1). Adaptive transfer of CD8 T cells into PD-1 KO mouse from wild type mice tolerated with TNP-apoptotic cells suppresses DTH. This result shows PD-1 functions on CD8 T cells for immune suppression. Additionally we neutralized the PD-1 with antibody to determine the phase when PD-1 functions for immune tolerance by apoptotic cells, and identified PD-1 functions particularly at the initial phase of antigen specific immune response. We are further studying the mechanism of suppressive role of PD-1+ CD8 T cells that should be activated with apoptotic cells.

